# Photobiomodulation therapy and endodontic treatment of teeth with apical periodontitis using 940-nm diode laser. Report of two cases

**DOI:** 10.4317/jced.59058

**Published:** 2022-03-01

**Authors:** Francisco Rubio, Federico Wienecke, Josep Arnabat-Domínguez, Pablo Betancourt

**Affiliations:** 1 Faculty of Dentistry, Universidad Andrés Bello, Concepción, Chile; 2 Department of Dentistry. Faculty of Medicine, University of Barcelona, Barcelona, Spain; 3 Endodontic Laboratory, Center for Research in Dental Sciences (CICO), Faculty of Dentistry, Universidad de La Frontera, Temuco, Chile; 4 Department of Integral Adultos; Faculty of Dentistry, Universidad de La Frontera, Temuco, Chile

## Abstract

**Background:**

Diode laser (DL) can be used in endodontics both for its bactericidal effect inside the root canal system (RCS) and for photobiomodulation therapy (PBMT) to accelerate the repair of periradicular bone tissue.

**Clinical Cases:**

This work presents two cases of pulp necrosis/asymptomatic apical periodontitis (AAP) that were treated with 940-nm DL, administered both to disinfect the root canal and to apply PBMT to the periradicular tissues. The cases were analysed by Cone-Beam Computed Tomography (CBCT).

**Discussion:**

DL has become widely accepted due to its high antimicrobial effectiveness and its ability to accelerate the repair of large apical lesions by biostimulation. Nevertheless, differences of opinion persist within the scientific community due to the lack of standardized endodontic protocols.

**Conclusions:**

The application of 940-nm DL, both for disinfection of the RCS and for PBMT, is an effective treatment in non-vital teeth with large periapical lesions. In both cases reported, bone neoformation were found at the 6-month check-up.

** Key words:**Low-level laser therapy, photobiomodulation therapy, diode laser, endodontics.

## Introduction

Apical periodontitis (AP) is an infectious inflammatory disease, prevalent all over the world, which increases with age (1). It occurs mainly as the result of infection of the root canal system (RCS), characterised by bone inflammation and destruction of the periradicular tissues, and may lead to tooth loss (2). The main goal in endodontics is the eradication of the microorganisms present in the whole RCS. This is extremely difficult, however, principally due to the morphological complexity of the tooth. Conventional treatments have proved insufficient to bring endodontic pathogenic microorganisms below detection limits, with success rates of no more than 62% to 83% in solving secondary/persistent infections (3). Thus new strategies are needed to control the bacterial biofilm and treat endodontic infections. In recent decades, interest in the use of diode laser (DL) to treat RCS has grown significantly, thanks to its affordability and broad spectrum of indications. Different studies have found that the energy released by DL in the root canal can exert a variety of effects, including photothermal disinfection and biostimulation of the periradicular bone tissue (4,5).

Photobiomodulation therapy (PBMT) applied with Low-Level Laser (LLLT) has shown good results recently in accelerating wound and apical cicatrization (6,7). The aim of this article was to report two cases of teeth with asymptomatic apical periodontitis (AAP) treated with PBMT and disinfection protocol by 940-nm DL.

## Case Report

**Figure 1:**
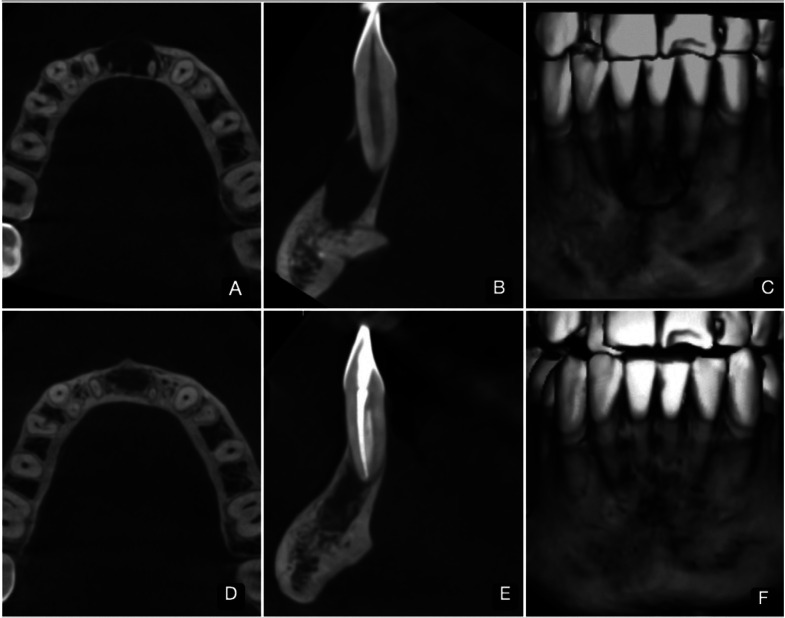
CBCT images of Clinical Case #1. A-B-C; Axial and sagittal view and three-dimensional reconstruction of the pre-treatment state. D-E-F; Axial and sagittal view and three-dimensional reconstruction of the post-treatment state after 6 months. Considerable diminution of the mesio-distal width can be observed, with peripheral areas of bone neoformation, recovery of lingual plate thickness and complete closure of the vestibular fenestration.

Case report 1. Male patient, 21 years old, with no relevant medical history. Panoramic and periapical X-ray examination showed an osteolytic lesion with mesio-distal width of 13.2mm, in the area of teeth 3.2-3.1-4.1-4.2, causing slight thinning of the lingual cortical plate and expansion and fenestration of the buccal plate (Fig. 1). Clinical examination showed a crown discoloration of tooth 3.1, soft tissues normal, percussion test and sensitivity thermal tests (ethyl chloride and guta-percha) negative. The diagnosis was pulp necrosis/AAP. Endodontic treatment of tooth 3.1 (single root canal) was carried out in 2 sessions. In the first session root canal instrumentation was carried out with a mechanised system #40/0.4 (Reciproc, VDW, Germany). After the use of each instrument, the root canals were irrigated with 1 mL of 2.5% NaOCl using a syringe and a 30-gauge side-vented needle (Becton Dickinson, Madrid, Spain) to the WL. The canals were irrigated with 1 mL ethylenediaminetetraacetic acid 18% (EDTA) (Ultradent, USA) for 1 min, followed by 1 mL of 2.5% NaOCl and 1 mL of saline. The irrigation protocol ended with the ultrasonic activation (Ultra X, Eighteeth, China) of NaOCl 5% for 60 sec, positioning the tip 2mm short to the working length.

**Figure 2:**
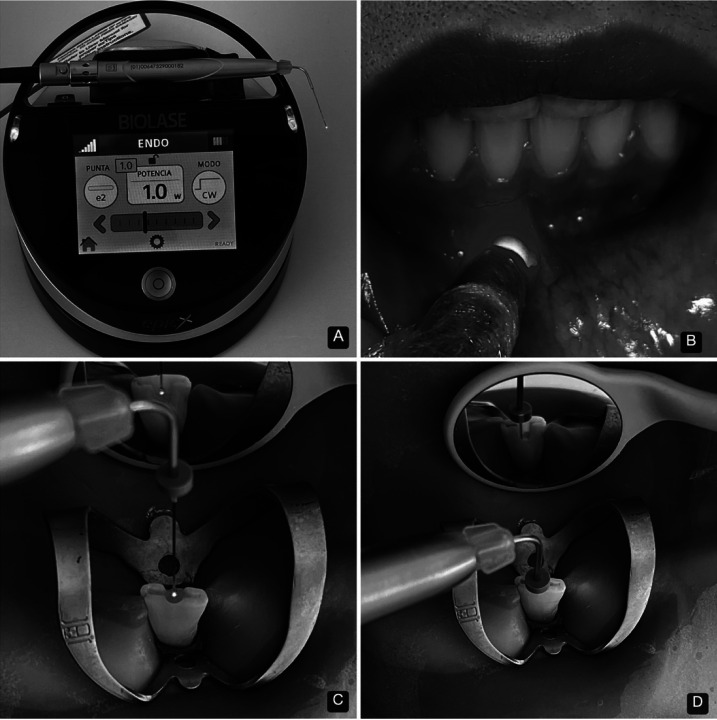
A; 940-nm diode laser unit used in endodontic disinfection and PBMT in the two cases reported. B; Clinical image of application of the PBMT protocol. C-D; Intracanal application of diode laser.

Dry intracanal disinfection was then performed with DL (940-nm) (EpicX, Biolase, USA), using 200 µm tips and power 1.0W. The tip was positioned -1mm from the work length, irradiating the root canal with an apico-coronal spiral movement at 1mm per sec, with 4 repetitions (10 sec between applications) (Fig. 2). Once dry, the canal was treated with calcium hydroxide (Ultracal XS, Ultradent, USA). PBMT was then applied with DL (940-nm) (EpicX, Biolase, USA), with one spot of 0.2 cm2,power 0.1W, for 40 sec per point, continuous mode, distributed in 4 points, using a total energy 16J in an area of 2.6 cm2, with an energy density 6 J/cm2 (Fig. 2). Surgical handpiece without tip was used for extraoral therapy.

The second session was carried out two weeks later. The same protocol of chemomechanical preparation (CMP) and laser disinfection were applied as described above. Root canal obturation was performed by the hydraulic method with bioceramic cement (Bioroot, Septodont, France). At the end of the first and second session, the same PBMT protocol was applied as described above. During the first week after completion of the endodontic treatment, one PBMT session was applied, followed by two in the second week and one each in the third and fourth weeks, making a total of 7 applications, all using the same protocol as described above. The defect volume and bone density were measured on CBCT images, initially and after 6 months post treatment (Fig. 1).

**Figure 3:**
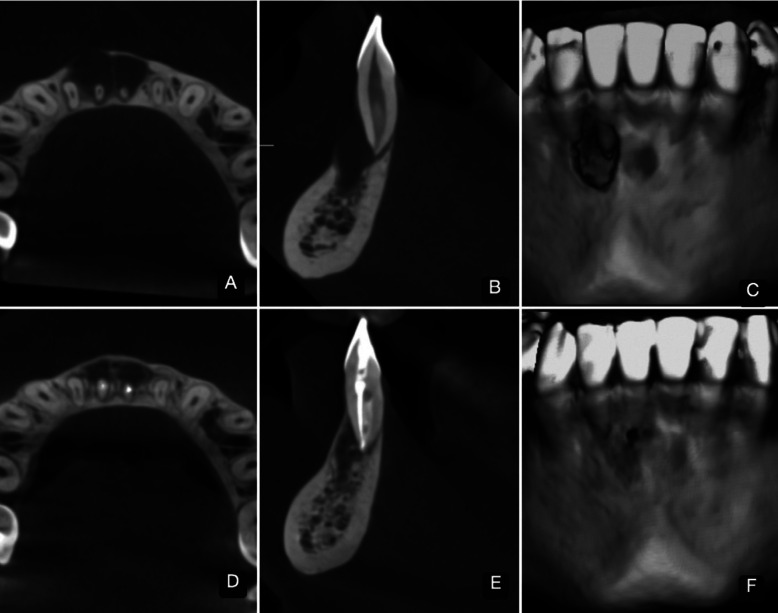
CBCT images of Clinical Case #2. A-B-C; Axial and sagittal view and three-dimensional reconstruction of the pre-treatment state. D-E-F; Axial and sagittal view and three-dimensional reconstruction of the post-treatment state after 6 months. A bony partition can be seen between the two hypodense focuses, with peripheral areas of bone neoformation and almost complete closure of the vestibular fenestration.

Case report 2. Female patient, 22 years old, with no relevant medical history, referred for assessment of apical radiolucency in teeth 3.2-3.1-4.1-4.2. Panoramic and pericapical X-ray examination showed a bilobulate osteolytic lesion, with mesio-distal width of 12.7mm, in the area of teeth 3.1 and 4.1, with expansion and fenestration of the vestibular cortical plate at tooth 4.1 (Fig. 3). Clinical examination showed soft tissues normal, percussion test and sensitivity thermal tests (ethyl chloride and guta-percha) negative. The endodontic diagnosis was pulp necrosis/AAP. Endodontic treatment of tooth 4.1 (single root canal) was applied in 2 sessions. The protocol used for CMP, disinfection of the root canal by DL and PBMT (7 applications in total) and root canal obturation was the same as described above for case #1. The defect volume and bone density were measured on CBCT images, initially and after 6 months post treatment (Fig. 3). The images were obtained on a Promax 3D CBCT unit (Planmeca, Helsinki, Finland), using 90 kV and 12 mA; FOV 8 × 8 cm, voxel size 0.15 mm, and analyzed with Romexis 4.5.1.R software (Planmeca, Helsinki, Finland).

## Discussion

The antimicrobial effect of DL is based on a direct photothermal effect on the pigmented membranes of the bacteria. DL at wavelengths of 810, 940 and 980 nm are hardly absorbed by water, allowing the light to interact with microorganisms located deep inside the dentin tubules. Conventional irrigation and ultrasonic activation are current techniques that present limitations in the disinfection of difficult-to-access areas, so the application of LLLT in endodontics seems to be a promising clinical alternative. Recently, Schulte-Lünzum *et al*. (8), using 940-nm DL with parameters recommended for endodontics (1–1.5W), observed a strong bactericidal effect against *Enterococcus faecalis* (83 to 99.99%), even at a depth of 1000μm, and with no adverse thermal effects. It should be noted that the thermal effect generated by DL can cause various complications inside the RCS if the parameters are not correctly regulated, such as root resorption and a change in dentin morphology (9). DL emits the energy in continuous wave (CW). However, CW in the root canal at higher power than 1.5 W is not advisable because of the heat damage that it may cause. It has been reported that if the laser tip comes into contact with the dentin wall during irradiation, it may cause heat damage, e.g. hot spots, fusion and cracks (9). For this reason, in the cases presented here we used a power of 1.0 W for disinfection of the RCS, based on endodontic protocols published previously (10). It has also been reported that the antimicrobial effectiveness of NaOCl is improved with 940-nm laser irradiation (10). It should therefore be noted that laser application is a complement to conventional CMP and not a substitute.

Postoperative pain is a frequent problem in endodontics. In the two cases reported here, the patients reported slight discomfort on the first day after treatment which diminished significantly on the second and third days. These observations agree with a recent study, in which postoperative pain diminished significantly on the third day after application of 940-nm DL in re-treatments (11).

PBMT uses low-intensity energy to induce cell proliferation and improve the differentiation of stem cells. Red or near-infrared light wavelengths are those most commonly used in PBMT (600-1100 nm) (12). It has been shown that the use of a fluence in the range of 0.04-50 J/cm2 is appropriate for generating optimum biological response (13). In the cases presented here, 6J/cm2 were applied, creating a biostimulating effect in both cases. It was shown that PBMT in a low energy density range accelerated repair in both cases, with bone neoformation, recovery of lingual cortical plate thickness and closure of the vestibular fenestration after 6 months. The explanation is that low-intensity energy stimulates release of the growth factors involved in the formation of epithelial cells, fibroblasts, collagen and vascular proliferation, as well as accelerating the synthesis of bone matrix due to increased vascularisation and reduced inflammatory response, with a significant increase of osteocytes in the irradiated bone (14). PBMT also appears to be a useful coadjuvant in the bone repair process, especially when associated with the use of bioceramics, since both accelerate the formation of bone tissue, promote the proliferation/maturation of osteoblast cells and accelerate bone regeneration (5).

A study using digital periapical radiography showed the effectiveness of LLLT repair through bone formation in cystic defects following cyst enucleation. After three months, the LLLT group presented a significantly greater increase in bone density than the control group (6). Metin *et al*. (7) evaluated the effect of LLLT on the cicatrization of hard and soft tissues after endodontic surgery, using CBCT. The authors observed significantly better results in terms of bone density, volume/area of the defect, and periapical index in the third month after the operation compared to the control group.

Nevertheless, not all reports have endorsed the use of LLLT in regenerative processes. Jakse *et al*. (15) found no positive effect of LLLT using DL at 680-nm (75mW) in a trial of bone regeneration and osseointegration of dental implants. These observations disagree with the results that we obtained in the two cases reported here. One possible explanation for the failure of the treatment was the laser application protocol, with only three sessions during the first week postoperative, low energy density (3-4 J/cm2) and the limited penetration of the wavelength used (680-nm); this dose is nowconsidered low for bone regeneration treatments. In contrast, the protocol used by us included a longer period of PBMT application after completion of the endodontic treatment, which may explain the differences between both studies.

## Conclusions

Based on the resolution of the cases, we can infer that the 940-nm DL effect could help accelerate the healing of the tissues affected by the AP. It should be taken into account that the application of LLLT is a complement to conventional endodontic therapy and not a replacement. To strengthen the evidence, further radomised clinical trial studies must be developed.
